# Marine Mammal Strandings and Environmental Changes: A 15-Year Study in the St. Lawrence Ecosystem

**DOI:** 10.1371/journal.pone.0059311

**Published:** 2013-03-27

**Authors:** Marie-Hélène Truchon, Lena Measures, Vincent L’Hérault, Jean-Claude Brêthes, Peter S. Galbraith, Michel Harvey, Sylvie Lessard, Michel Starr, Nicolas Lecomte

**Affiliations:** 1 Département de Biologie, Chimie et Géographie, Université du Québec à Rimouski, Rimouski, Québec, Canada; 2 Department of Fisheriesand Oceans, Maurice Lamontagne Institute, Mont-Joli, Québec, Canada; 3 Institut des Sciences de la Mer, Université du Québec, Rimouski, Québec, Canada; 4 Department of Environment, Government of Nunavut, Igloolik, Nunavut, Canada; Institute of Marine Research, Norway

## Abstract

Understanding the effects of climatic variability on marine mammals is challenging due to the complexity of ecological interactions. We used general linear models to analyze a 15-year database documenting marine mammal strandings (1994–2008; n = 1,193) and nine environmental parameters known to affect marine mammal survival, from regional (sea ice) to continental scales (North Atlantic Oscillation, NAO). Stranding events were more frequent during summer and fall than other seasons, and have increased since 1994. Poor ice conditions observed during the same period may have affected marine mammals either directly, by modulating the availability of habitat for feeding and breeding activities, or indirectly, through changes in water conditions and marine productivity (krill abundance). For most species (75%, n = 6 species), a low volume of ice was correlated with increasing frequency of stranding events (*e.g.* R^2^_adj_ = 0.59, hooded seal, *Cystophora cristata*). This likely led to an increase in seal mortality during the breeding period, but also to increase habitat availability for seasonal migratory cetaceans using ice-free areas during winter. We also detected a high frequency of stranding events for mysticete species (minke whale, *Balaenoptera acutorostrata*) and resident species (beluga, *Delphinapterus leucas*), correlated with low krill abundance since 1994. Positive NAO indices were positively correlated with high frequencies of stranding events for resident and seasonal migratory cetaceans, as well as rare species (R^2^_adj_ = 0.53, 0.81 and 0.34, respectively). This contrasts with seal mass stranding numbers, which were negatively correlated with a positive NAO index. In addition, an unusual multiple species mortality event (n = 114, 62% of total annual mortality) in 2008 was caused by a harmful algal bloom. Our findings provide an empirical baseline in understanding marine mammal survival when faced with climatic variability. This is a promising step in integrating stranding records to monitor the consequences of environmental changes in marine ecosystems over long time scales.

## Introduction

Environmental changes are occurring worldwide and the consequences of recent climatic variability are now acknowledged as a global perturbation with geographic differences in intensity [1]. Spatial heterogeneity in degree of environmental change and climatic variability poses a challenge in monitoring effects on terrestrial and marine communities [2], [3], [4]. This is especially true for top predators, such as marine mammals, because their position in food webs and long generation time make them especially sensitive to perturbations in ecosystems [2], [5]. Marine mammals are threatened at the global scale with almost a quarter of these species on the verge of extinction [6], [7].

**Figure 1:**
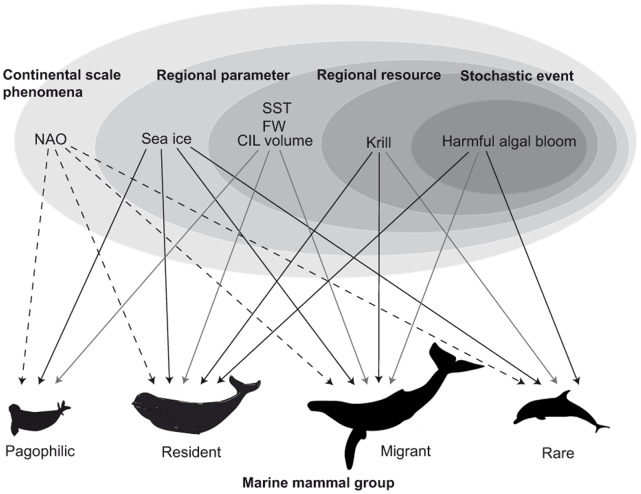
Conceptual framework showing how environmental parameters may affect stranding events of marine mammals in the Estuary and the Gulf of St. Lawrence, Québec, Canada. Black solid lines are possible effects of regional parameters and dashed lines are possible indirect effects of continental parameters identified in this study, while grey lines are additional effects previously reported in the literature (see Table 1).

Stranding records have long been used as an indirect means to monitor the status, distribution, and seasonal abundance of marine mammals [8], [9], [10]. These records have also been instrumental in detecting unusual mortality events [11], [12], [13]. A growing number of studies have identified factors directly affecting individual survival such as human activity, together with those affecting post-mortem drift such as currents [14], [15], [16]. Recent work has corroborated inter-annual variation in long-term stranding data with climatic variability, demonstrating the utility of stranding events as real-time bio-indicators [8], [17]. To understand complex (and variable) ecological responses of marine mammals to environmental change, we need to consider environmental parameters over multiple scales (regional and continental) and to address data from multiple species [17], [18]. Such a hierarchical approach provides a promising avenue to evaluate effects of climatic variability; this is especially true when dealing with species with great variability in habitat use, *e.g.* with resident *vs.* migratory species (Fig. 1) [19], [20].

The St. Lawrence ecosystem, in eastern Canada, is an important feeding area for numerous marine mammals of the North Atlantic Ocean, including several species at risk [*e.g.* endangered blue whale (*Balaenoptera musculus),* endangered North Atlantic right whale (*Eubalaena glacialis),* and threatened St. Lawrence Estuary beluga (*Delphinapterus leucas*)]. Recent environmental changes in the St. Lawrence (*e.g.* below average ice cover over the last decade) and structural changes in the marine community (*e.g.* composition, abundance, and distribution of several invertebrates and fishes) have been identified as critical factors affecting the dynamics of marine mammal populations [21], [22], [23]. The systematic record of marine mammal stranding events since 1994 can be used as a proxy of natural mortality over such a large ecosystem. This provides a unique opportunity to identify the temporal variability of oceanographic changes affecting a complex community of marine mammals.

Here, our main objective was to investigate possible relationships between temporal variation in marine mammal stranding events and changes in environmental conditions occurring in the St. Lawrence Estuary (SLE) and the Northwestern Gulf of St. Lawrence (NWGSL) from 1994−2008 (Fig. 1). Recently, low ice cover was linked to negative North Atlantic Oscillation index values (NAO), which is a proxy for climatic variability over the whole North Atlantic Ocean. This change in ice cover is a likely mechanism to explain the increase in young-of-the-year harp seal (*Pagophilus groenlandicus*) mortality in the Gulf of St. Lawrence [17]. This is an example of hierarchical effects of environmental parameters, though it remains unclear whether such links are affecting other species in the same manner.

We hypothesized that key environmental parameters (listed in Table S1 in File S1) affecting stranding events such as ice volume, have a different impact on marine mammal survival depending on their dispersal strategy (*i.e.* resident *vs.* seasonal migrant) and habitat use (*i.e.* for breeding *vs.* for feeding). Specifically, we predicted that regional parameters measured with CIL volume and sea temperature (low values) will negatively influence resident species (high number of strandings). We also predicted that sea ice volume (low values) will negatively influence stranding numbers of pagophilic species (high number of strandings), and krill abundance (low values) will negatively influence stranding numbers of mysticetes species (high number of strandings). Finally, we predicted that NAO index (low index), known as a key large-scale continental parameter, will influence positively migrants species (low number of strandings) but negatively pagophilic species (high number of strandings).

## Methods

### Ethic Statement

Field-work endeavors in the marine ecosystems of Canada were subject to approval by the Department of Fisheries and Oceans Canada (DFO). Co-authors Michel Harvey (DFO) and Michel Starr (DFO) were responsible of krill and phytoplankton sampling, respectively, and notified DFO with their work. For the purpose of this paper, we did not need additional permits as we used an existing database recorded under the jurisdiction of the DFO.

### Study Area

**Figure 2:**
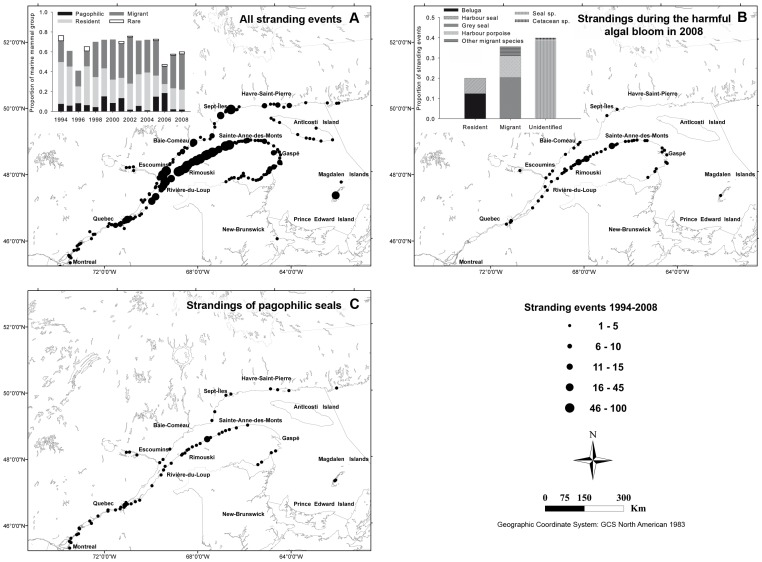
Study area and distribution of marine mammal stranding events in the Estuary and the Gulf of St. Lawrence, Québec, Canada, 1994–2008. (A) All reported marine mammal stranding events (1994–2008), (B) stranding events reported only during the harmful algal bloom of 2008, and (C) stranding events of pagophilic seals only (1994–2008). Marine mammal groups used in analyses are defined in Table S2 in File S1.

Marine mammal stranding events were documented along the Northwestern shores of the Gulf of St. Lawrence (NWGSL) and the St. Lawrence Estuary (SLE), Québec, Canada (Northwest Atlantic Fisheries Organisation Divisions 4S and 4T) (Fig. 2A). The St. Lawrence is a complex and dynamic ecosystem receiving great quantities of salt and fresh waters [24]. Persistent high-density aggregations of krill and fish (*i.e.* capelin) make the SLE [25], [26] and the NWGSL important feeding areas for a variety of marine mammal species in summer [27].

During winter, the cold (<−1°C) surface mixed layer reaches an average depth of 75 meters and encompasses up to 45% of all the waters of the Gulf of St. Lawrence [28]. Spring surface warming, sea-ice melt and continental runoff produce a surface layer below which the winter-formed cold layer is partly isolated from the atmosphere. This forms the summer cold intermediate layer (CIL), which persists until the next winter, gradually warming up and deepening during summer [29], [30].

### Collection of Stranding Data

We assembled all available data on marine mammal stranding events held by DFO and the stranding network *Réseau québécois d’urgences pour les mammifères marins* (RQUMM). Annual awareness campaigns provided information about the stranding network to numerous organisations and citizens, who were invited to report stranding observations. From 1994 to 2002, stranding data was collected by DFO at the Maurice Lamontagne Institute (Qc, Canada) and since 2003, by RQUMM, a marine mammal response network co-ordinated by the non-governmental organization, *Groupe de recherche et d’éducation sur les mammifères marins* (GREMM) under contract to DFO. We systematically examined these two databases for both single and mass stranding events, the latter defined as at least two animals ashore at the same place and time except for cases involving a female and calf which are considered single events [31]. Floating carcasses and strandings attributed directly to human activity were excluded (N = 192, 16% of total stranding measured in this study). Evidence of human incidence on strandings was based on specific criteria and was classified by categories (*e.g.* the by-catch category was identified when the fishing gear was found on the animal or when unhealed, narrow, linear lacerations or indentations were observed on the epidermis of the carcass). These data are the object of a manuscript in preparation (Truchon *et al.* pers. comm.). Analyses of stranding data included only cases where identification of marine mammal species was confirmed (photographs and/or trained observers were used to confirm species identification). Ambiguous cases of species identification were classified as “seal spp.” or “cetacean spp.” and were excluded from single species analyses. Seasons were defined as Spring (Sp) - March to May, Summer (S) - June to August, Fall (F) - September to November and Winter (W) - December to February.

### Environmental Data

Environmental data were obtained from various DFO and National Oceanic and Atmospheric Administration (NOAA) monitoring programs. Data included environmental parameters involving linkages between environmental changes and marine mammals (see Table 1, Fig. 1, and below for details) at regional and continental scales. The parameters for the regional scale are: (1) abundance of some species of harmful toxic algae (TA) (St. Lawrence Global Observatory: http://ogsl.ca/en/phytoplankton.html), (2) krill abundance (Krilla) [22], [32], (3) sea surface temperature (SST) [33], (4) freshwater runoff (FWR), http://ogsl.ca/en/runoffs/data/tables.html), (5) volume of the cold intermediate water layer (CIL) (Vol0) [23] and (6) volume of sea ice cover (ICEV) [23]. Finally, parameters for the continental scale are: (1) North Atlantic Oscillation (NAO) index of the current year, (2) NAO index of the previous year (NAO_t–1_), and (3) NAO index of the previous winter (NAO_wt–1_).

**Table 1:** Studies documenting ecological linkages between marine mammals and environmental changes.

Marine mammals and environmental factors	Temporal scale (years)	Type of data used	Literature cited
Cetacean and thermal fronts	<10	Sighting data	[59], [60]
Cetacean and sea ice	<10	Sighting data	[2], [53]
Cetacean and large-scale climatic factors	>10	Sighting and stranding data	[3], [20]
Cetacean and harmful algal blooms	<10	Stranding data	[13], [63]
Cetacean and ressource availability	<10	Sighting data	[26], [64]
Pinniped and thermal fronts	<10	Diving data	[56], [60]
Pinniped and sea ice	>10	Demographic and stranding data	[17], [39], [40]
Pinniped and large-scale climatic factors	>10	Demographic and stranding data	[17], [39]
Pinniped and harmful algal blooms	>10	Stranding data	[12], [63]
Pinniped and resource availability	<10	Demographic and diving data	[55], [56]
Rare cetacean species and water conditions	55	Stranding data	[49]
Climate change and marine mammals	*-*	This study and reviews	[3], [6]

This table is limited to selected citations given as examples, and does not constitute an exhaustive review of the literature. This table can be linked to Fig.1.

#### Regional environmental data

Annual mean of harmful toxic algae abundance was recorded at eleven coastal stations covering the SLE and GSL. Phytoplankton samples were taken every week from mid-May to late October (see [34] for details). We selected and pooled seven taxa with potential toxic effects: *Alexandrium* spp., *Dinophysis* spp., *Karenia mikimotoi, Prorocentrum lima, Prorocentrum minimum, Pseudo-nitzschia pseudodelicatissima,* and *Pseudo-nitzschia seriata.*

Annual index of estimated krill abundance included three species (*Meganyctiphanes norvegica*, *Thysanoessa raschii,* and *Thysanoessa inermis*) and were collected at four fixed stations, in mid-June and late fall (late October and early November). Zooplankton collections and standard measurements are outlined in [35] and were corrected (see [22] for details). SST was averaged over the entire GSL (195 000 km^2^) for each year (1994 to 2008) between June to August, inclusively, when marine mammals most frequented the area (see [23] for details of the methods and [33] for recent inter-annual variability over the GSL). For other parameters, we used mean annual freshwater runoff (FWR) measured at Québec City (main freshwater input), August/September volume of the CIL at T<0°C and mean annual volume of sea ice cover of the entire GSL (obtained from Canadian Ice Service daily digitized ice charts where standard thicknesses are attributed to ice types) including breeding areas of pagophilic seals (harp and hooded seals).

#### Continental environmental data

To assess the effect of large-scale climatic variability on stranding numbers at a continental scale, we used NAO index values obtained from the Climate Prediction Center of the National Weather Service (www.cpc.ncep.noaa.gov). NAO data are calculated using the difference in sea level pressure between Lisbon, Portugal and Stykkisholmur/Reykjavik, Iceland. The amplitude of the NAO index is known to have a major effect on ice conditions in Arctic and Atlantic Oceans, including a time-lag effect from the previous year and previous winter on the current year [36], [37], [38]. The NAO index consists of a north-south dipole of air pressure anomalies with one center located over Greenland and the other center covering the central latitudes of the North Atlantic Ocean, between 35″N and 40″N latitudes [39]. Several studies link the recruitment of pagophilic species *(i.e.* ringed seal, *Phoca hispida* and harp seal) to the current year NAO index, the NAO index of the previous year (NAO_t–1_), the NAO index of the previous winter (NAO_w t–1_, measured as the NAO index from December to March of the previous year), and ice conditions [39], [40]. We included all these parameters in our analyses.

### Statistical Analyses

All statistical analyses were conducted with R.2.8.0 [41]. Analyses of variance (ANOVAs) were first used to detect differences in the occurrence of stranding events between years and seasons followed by post-hoc t-tests for single factors [42]. Kruskall-Wallis tests were used when Shapiro-Wilkinson tests indicated failure to meet the two assumptions of normality and homogeneity of variance, thereby precluding the use of parametric tests as well as mathematic transformations [42]. Differences were statistically significant at p<0.05.

Multiple linear regressions were performed to model the relationships between stranding events (*e.g.* response variable) and environmental parameters (*e.g.* explanatory variables) by species. All models were tested with Shapiro-Wilkinson tests to meet the two assumptions of normality and homogeneity of variance. To overcome the variability of sampling effort, we used de-trended stranding data by using residuals in multiple linear regression models. For pagophilic seal species, breeding within the area during winter, and for resident species, two different models were used based on species (Table S1 in File S1); NAO index of the previous year (NAO_t–1_), and the other with NAO of the previous winter (NAO_wt–1_), respectively. Akaike’s Information Criterion (AIC) was used to select the most parsimonious model [43], unless the differences in AIC were smaller than 2 in which case the best explicative model was selected (highest values for their adjusted R^2^).

We restricted our models to exclude collinear variables by using a Pearson’s correlation coefficient greater than 0.6. To avoid problems in regression fitting caused by the strong correlation of ice cover volume with krill abundance (0.7) and CIL volume (0.6), we used the residuals of ice cover volume (resICEV), indicating the deviation from average, as a predictor [44]. We then obtained nine predictors (Vol0, SST, FWR, resICEV, krilla, TA, NAO, NAO_t–1_ and NAO_wt–1_), which were not correlated. The most parsimonious model was compared to an intercept-only model (*i.e.* without predictor variables) to evaluate the power of environmental parameters selected for each single species model.

## Results

From 1994 to 2008, a total of 1,193 stranding events were reported on the shores of the St. Lawrence, and included 549 cetaceans (405 odontocetes, 96 mysticetes, and 48 cetacea spp.) and 644 seals (260 identified to the species level, see Table S2 in File S1). Cetacean strandings included 10 and 5 species of odontocetes and mysticetes, respectively. Belugas (n = 205) and minke (n = 61) whales were the most frequently reported odontocetes and mysticetes, respectively. Seal strandings included five species, with harbour (*Phoca vitulina*, n = 80) and grey (*Halichoerus grypus*, n = 80) seals being the most frequently reported. Although the large majority of stranding events involved single individuals, a total of 22 mass stranding events were reported for the Magdalen Islands (Fig. 2.A; 41% of 54 reports). Mass stranding events involved mostly harp seals (n = 5, representing 53% of total number of individuals involved in seal mass strandings), Atlantic White-sided dolphins (*Lagenorhynchus acutus*) (n = 5), hooded seal (n = 2), and unidentified seals (n = 15). During the study period, there were very few stranding observations involving rare or less common species in the SLE (n = 16; see Table S2 in File S1).

### Temporal Patterns

**Figure 3:**
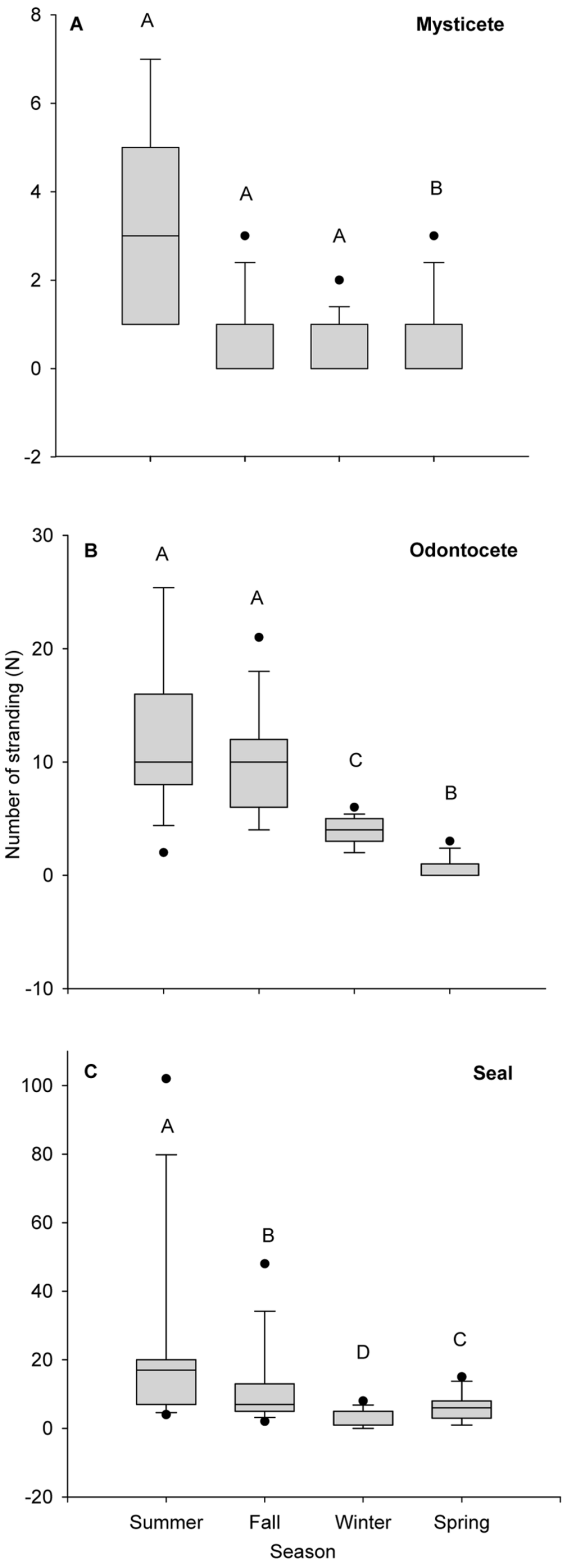
Seasonal variability in the mean number of marine mammal stranding events in the Estuary and the Gulf of St. Lawrence, Québec, Canada, 1994–2008. (A) Mysticetes, (B) odontocetes and (C) seal species in the Estuary and the Gulf of St. Lawrence, Quebec, Canada, 1994–2008. Letters over the bars indicate statistical differences (see methods); bars with different letters are statistically different at 5% level.

Overall, stranding reports were more frequent during summer and fall compared with spring and winter (odontocetes: *F*_3,179_ = 56.74, *p*<0.001, n = 405; mysticetes: χ^2^ = 37.02, df = 3, *p*<0.001, n = 96; and seals: *F*_3,179_ = 33.53, *p*<0.001, n = 644; Fig. 3).

De-trended stranding data increased during the study period (r = 0.77). This trend was possibly driven by the low number of strandings recorded at the beginning of the time series (1994–1998) followed by high numbers reported from 1999 to 2007 as well as in 2008 when the harmful algal bloom occurred. The increasing trend remained even after excluding 2008 (large standard deviation for the number of stranding events caused by the harmful algal bloom in summer 2008; see below).

### 2008 Event

A multi-species stranding event occurred in August 2008, involving grey seals, harbour seals, harbour porpoises (*Phocoena phocoena*), and belugas (Fig. 2.B). During this event, a total of 114 carcasses (62% of the year’s total) were found on the south shore of the SLE. Grey seal stranding numbers were numerous (n = 35), being 122 times the 14-year mean (1994–2007) of August 2008. Concurrently, beluga stranding numbers (n = 10) amounted to three times the mean numbers documented for that single month from 1983 to 2007 (DFO, unpublished data).

Necropsies performed on carcasses indicated that most animals were in good nutritional condition with no lesions attributable to any infectious, parasitic or other etiological agent (DFO, unpublished data). However, analyses of tissues from marine mammal carcasses (including belugas, harbour porpoises, harbour and grey seals), invertebrates, fishes, and birds revealed the presence of biotoxins produced by *A. tamarense* (DFO, unpublished data).

### Environmental Explanatory Parameters

**Figure 4:**
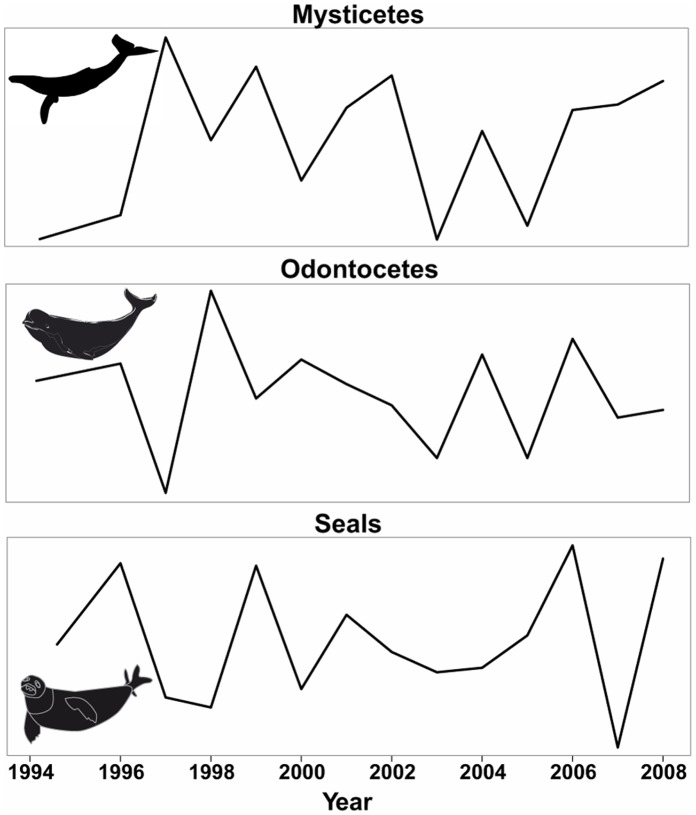
Inter-annual variation of marine mammal stranding events in the Estuary and the Gulf of St. Lawrence, Québec, Canada, 1994–2008. The time series are de-trended (see methods) to indicate inter-annual variation rather than long-term changes and remove any potential bias due to increased observation reports over the years.

Multiple linear regression models were performed on de-trended data from the period 1994–2008 (Table S3 in File S1 and Fig. 4). Results showed that inter-annual changes in environmental parameters significantly affected the occurrence of stranding events of five marine mammal species (*i.e.* models significantly differed from the null models, *p*<0.05; Table 2). Environmental parameters failed to explain inter-annual variations of stranding events for two species (*i.e.* harbour seal and fin whale, *Balaenoptera physalus*), and one species (harp seal) was not included as the Shapiro-Wilkinson test indicated failure to meet the assumption of normality. The explanatory power of models including several environmental parameters varied greatly based on the species (ranging from 34% to 81% for the best models, Table 2). The models including minke whale and hooded seal stranding numbers as response variables had the best predictive power (81% and 59%, respectively).

**Table 2:** Multiple linear regression model coefficients for environmental parameters tested as predictors of inter-annual variation in marine mammal stranding events in the Estuary and the Gulf of St. Lawrence, Québec, Canada, 1994−2008.

Parameter^a^	Resident^b^	Seasonal migrant^c^				Pagophilic^d^	Seal^e^ mass	Rare species^f^
	Dl	Pp	La	Ba	Hg	Cc	stranding	
SST		−2.79		0.7		9.22**		
Vol 0	9.48×10^−4^*			3.36×10^−4^		−0.002*		−3.46×10^−4^Δ
resICEV			−5.87×10^−11^	−2.27×10^−10^ *	−3.83Δ	−1.59×10^−10^	−5.86×10^−11^Δ	−9.20Δ
FWR	−8.81×10^−4^	−0.002				0.002	3.23×10^−4^	6.63×10^−4^*
Log. Krilla	−5.29Δ			−3.14Δ				
Log.TA		6.71 *		3.34 *	6.24			2.38*
NAO			3.92 *	4.04**	−1.78*			
NAO _t–1_			−2.61Δ	2.39				
NAO_wt–1_	5.43**				1.20*		−1.37*	1.12
*p*	0.02	0.04	0.03	0.004	0.05	0.009	0.0002	0.1
Adj. R^2^	0.53	0.40	0.41	0.81	0.41	0.59	0.40	0.34

a Parameters in most parsimonious models : sea surface temperature (SST, °C), cold intermediate layer volume at 0 °C (Vol0, km^3^), ice cover volume residuals (resICEV, Km^3^), freshwater runoff (FWR, 10^3 ^m^3^s^−1^), abundance of krill (Krilla, ind. m^−3^), abundance of harmful toxic algae (TA, cell L^−1^), North Atlantic Oscillation (NAO) index, NAO index of the previous year (NAO _t–1_) and NAO index of the previous winter (NAO_wt–1_).

b Resident species is beluga whale (*Delphinapterus leucas*, Dl),

c Seasonal migrant species are Harbour porpoise (*Phocoena phocoena*, Pp), Atlantic White-sided dolphin (*Lagenorhynchus acutus*, La), Minke whale (*Balaenoptera acutorostrata*, Ba) and Grey seal (*Halichoerus grypus*, Hg),

d Pagophilic species is Hooded seal (*Cystophora cristata*, Cc) and,

e Seal and

f Rare species as listed in Table S1 in File S1. Δp<0.1; *p<0.05; **p<0.01.

#### Regional parameters

**Figure 5:**
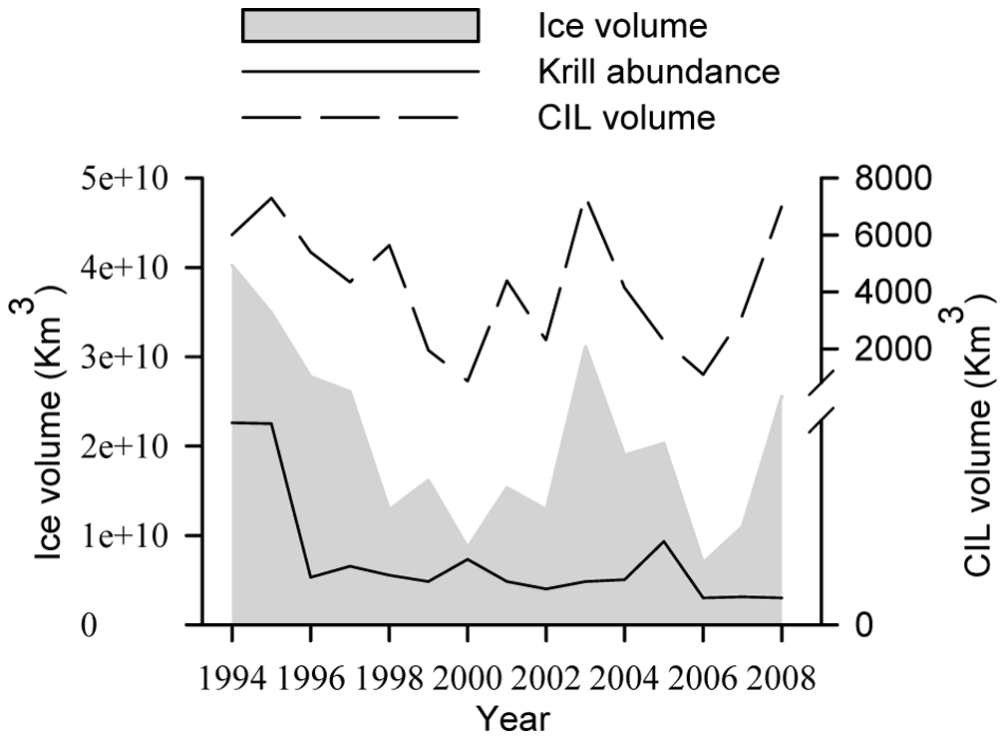
Retrospective variations of ice cover volume, CIL volume in the Gulf of St. Lawrence, and krill abundance in the St. Lawrence Estuary (1994 to 2008), Québec, Canada.

Two of the eight parameters were negatively correlated with years; ice cover volume and krill abundance (r = −0.51, *p = *0.005; r = −0.72, *p* = 0.002, respectively). Ice cover volume was also positively correlated with krill abundance and CIL volume (r = 0.66, *p* = 0.007; r = 0.78, *p*<0.001, respectively; Fig. 5). Water parameters in general, including CIL volume and SST, were recurrently selected in models with species-specific relationships (Table 2). Overall, years with low ice conditions positively affected stranding events for all species (higher number of strandings), except for the harbour porpoise and resident beluga. Relationships between marine mammal stranding events and other environmental parameters were species-dependent. With respect to biotic factors, years with low krill abundance were correlated with high stranding events of belugas and minke whales. In addition, the multi-species stranding event in August 2008 coincided with a harmful algal bloom of *A. tamarense* in the SLE.

#### Continental parameters

The NAO index positively affected stranding numbers of two migrant species (*i.e.* Atlantic White-sided dolphin and minke whale) compared to the grey seal (Table 2). Stranding numbers of the Atlantic White-sided dolphin and the minke whale were negatively and positively correlated with the previous year NAO index, respectively. Stranding numbers of resident and migrant species (*i.e.* belugas, grey seals together with rare species) were positively correlated with the previous winter NAO index. In contrast, seal mass strandings were negatively correlated with the previous winter NAO index.

## Discussion

Here we identify potential links between stranding events and environmental conditions using a multiple species and scale approach. Our multiple regression models suggest that the inter-annual variability of marine mammal stranding events over a 15-year period is associated with regional (*e.g.* ice volume) and continental (*e.g.* NAO index) changes in the environment for both resident and migrant species (Table 2). The marine mammal community was likely affected by low ice conditions observed in recent years through two pathways; 1- directly with changes in ice conditions modulating the availability of habitat for feeding and breeding activities or 2- indirectly with changes in water conditions and marine productivity (*e.g.* using krill abundance as an index). Moreover, the magnitude and timing of a stochastic event, the harmful algal bloom in August 2008, at the head of the Laurentian Channel, an area of upwelling in the St. Lawrence Estuary, affected migrant and resident species.

### Implication of Sea Ice Changes

In the temperate ecosystem of the St. Lawrence, ice is a fundamental component structuring the marine mammal community by creating habitats for some species (*e.g.* whelping substrate for pagophilic phocids in winter [40], [45]) and acting as a potential barrier for some others [46]–[47]. Ice volume is high during severe winters, which also favours a high CIL volume for the following summer, regionally affecting krill abundance and patchiness [23], [26], [28].

Over the last decade, poor ice conditions have been observed compared to ice conditions in the late 1980 s and 1990 s in eastern Canada. In some years, neonatal mortality of the North Atlantic harp seal has been documented in association with poor ice conditions [40], [45]. Our models are consistent with previous reports of pagophilic seal mortality [39], [40], [45] as we demonstrated an association between reduced ice cover volume and increased strandings of single hooded seals and mass strandings of seals (particularly juveniles, with harp seal representing 53% of the total number of individuals involved in seal mass stranding events). Large-scale climatic parameters such as NAO and NAO_w(t−1)_ indices are known to modulate sea ice and consequently affect the survival of pagophilic seal pups during the whelping season [17], [39]. Here, we also found an association between negative NAO phase, *i.e.* last winter (NAO_w(t−1)_) index, and increased mass mortalities of seals, with mortality correlated with low ice cover volume.

Environmental changes may also drive shifts in composition and structure of marine communities with climatic variability directly affecting organisms or indirectly through the food web, from primary to secondary producers [48], [49]. Our models support previous studies that local prey resources are important (using krill abundance as a proxy) for resident species (*e.g.* beluga) and migrant mysticete species (*e.g.* minke whale) using the SLE and GSL for feeding activities (*i.e.* particularly in areas of upwelling where krill and fish are aggregated along continental shelves) [25], [26]. While the decline of krill abundance in the St. Lawrence since 1994 has not been thoroughly investigated yet [22], [50], our results suggest a relationship between low krill abundance and poor ice conditions observed in the 2000 s, which has likely affected the marine mammal community. The correlations found between decreased krill abundance, low CIL volume and low ice cover volume suggest that recent low ice cover volume affected resource availability through water conditions, with krill abundance specifically associated with CIL volume [22], [26]. Beside this, we suspect the negative relationship found between ice volume and number of strandings of migrants species results from the release of ice barriers and distribution changes in winter and early summer [51]; low ice volume into the Gulf could yield new ice-free habitats for migrant species [52], [53] and allow movement toward the Gulf and Estuary -this would increase the odds of stranding events in our study area (*e.g.* increase odds of sporadic mortality such as in ice entrapment events [46], [47]. Alternatively, when sea ice volume is low, a sick animal or a carcass may be more able to get to the shore to strand than when sea ice is consolidated. Nevertheless, a carcass entrapped in sea ice can drift and strand ashore with a delay driven by the timing of spring melt or during winter warming periods that can precipitate the thinning of sea ice. However, a seasonal delay in stranding numbers is not expected to drive the negative relationship between sea ice volume and stranding numbers because we considered the total annual stranding numbers in all models. Moreover, most cetacean species feeding in the study area during the summer time leave the area in fall, avoiding harsh winter conditions. Therefore this migration pattern reduces the likelihood of carcasses being entrapped in sea ice within our area [54].

Large-scale climatic parameters such as NAO and NAO_t−1_ are also important in models for migratory species such as the Atlantic White-sided dolphin and the minke whale. This is consistent with NAO indices affecting migratory behavior, body condition and survival of marine mammals indirectly through effects on local resource availability [55], [56].

### The Use of Stranding Data and the Multi-scale Approach

In general, temporal variability in oceanographic conditions is particularly high in temperate ecosystems where seasonal ice plays a major role in the dynamics of marine populations [20]. This seasonal variability may reduce our ability to detect and interpret ecological mechanisms affecting marine populations due to climatic variability, particularly for migratory species [20]. In this context, the use of long-term stranding datasets allows the identification of possible ecological mechanisms between climate variability and marine mammal communities (review in Table 1). The multi-scale (regional and continental) and multi-species datasets used in this study illustrate the complexity, underlying seasonality, various pathways through the food web (*e.g.* the relationship between ice, krill abundance and migrants), as well as the effect of stochastic events (*e.g.* the harmful algal bloom in 2008) (Fig. 1). Although marine mammals are highly mobile and can avoid adverse local conditions, harmful algal bloom events in habitats where animals feed increase the probability of intoxication and mortality. In the Estuary, harmful algal blooms are a recurrent phenomenon known to occur in many restricted areas at the mouth of rivers and are associated with above-normal freshwater runoff during summer [57], [58], but have not, to our knowledge, been recorded in an area with upwelling (*i.e.* the head of the Laurentian Channel, described in [26]) leading to unusually high mortality of several marine species as observed in 2008. As the toxic dinoflagellate, *A. tamarense* is sensitive to some environmental changes [58]; monitoring harmful algal blooms promotes protection and conservation of marine mammals as well as public health.

Stranding data do not allow us to define the ecological mechanisms underlying changes in water conditions (*e.g.* SST and FWR). In contrast, the effect of water conditions (*e.g.* fronts, eddies, topography) on widely distributed cetaceans has largely been examined at a fine spatio-temporal scale, focusing on marine mammal sighting data rather than stranding data [59], [60]. As a way forward, we suggest that sighting data may be combined in the future with stranding data as a more comprehensive tool to understand ecological linkages [61]. This would be especially relevant in a feeding area where water parameters affect prey aggregation and consequently the distribution of marine mammals into predictable areas [59] (Table S1 in File S1).

Despite inherent limitations associated with marine mammal stranding data, we demonstrated the importance of using a multiple scale and species approach to detect unexpected ecological linkages compared to mono-specific or single scale studies [17], [62]. This approach has been useful in identifying relatively complex ecological linkages (*e.g.* distribution of migrant whales in winter with respect to ice conditions). Overall, its uses may help in designing future researches on the effect of climatic variability on the diverse and vulnerable community of marine mammals.

## Supporting Information

File S1 **File contains: Table S1.** Species composition in the stranding database for the Estuary and the Gulf of St.-Lawrence, Québec, Canada, 1994–2008. This table provides an overview of all marine mammal species stranded in the study area. Age and sex classes for each species were grouped together. Table adapted from [54]. **Table S2.** Species composition of marine mammal strandings reported in the Estuary and the Gulf of St.-Lawrence, Québec, Canada, from 1994–2008 (N = 1193). **Table S3.** Model selection for multiple linear regressions including environmental parameters as predictors of the inter-annual variation in marine mammal stranding events in the Estuary and the Gulf of St. Lawrence, Québec, Canada, 1994–2008. For each model, we report the sample size (n), the number of parameters (k), the Akaike Information Criterion (AIC), the value relative to the model with the lowest AIC (ΔAIC), AIC weight (ωAIC) as well as the adjusted R^2^ (R^2^adj.). Models are ranked by their AIC and best models are shown in bold.
(DOC)

## References

[1] Intergovernmental Panel on Climate Change (IPCC) (2007), Climate Change 2007: The Physical Science Basis. Contribution of Working Group I to the Fourth Assessment Report of the Intergovernmental Panel on Climate Change, edited by S. Solomon et al., Cambridge Univ. Press, Cambridge, U. K., Available:http://www.ipcc.ch/publications_and_data/publications_and_data_reports.shtml#1. Accessed 16 January 2013.

[2] Moore SE (2008) Marine mammals as ecosystem sentinels. J of Mammal 89: 534–540.

[3] Evans PGH, Pierce GJ, Panigada S (2010) Climate change and marine mammals. J Mar Biol Assoc UK 90: 1483–1487.

[4] Post E, Forchhammer MC, Bret-Harte MS, Callaghan TV, Christensen TR, et al (2009) Ecological dynamics across the Arctic associated with recent climate change. Science 325: 1355–1358. 19745143 10.1126/science.1173113

[5] Carbone C, Gittleman JL (2002) A common rule for the scaling of carnivore density. Science 295: 2273. 11910114 10.1126/science.1067994

[6] Schipper J, Chanson JS, Chiozza F, Cox NA, Hoffmann M, et al (2008) The status of the world's land and marine mammals: diversity, threat, and knowledge. Science 322: 225–230. 18845749 10.1126/science.1165115

[7] IUCN (2011) IUCN Red List of Threatened Species. Version 2011.2. Available: www.iucnredlist.org. Accessed 30 May 2012.

[8] Evans K, Thresher R, Warneke RM, Bradshaw CJA, Pook M, et al (2005) Periodic variability in cetacean strandings: links to large-scale climate events. Biol Letters 1: 147–150. 10.1098/rsbl.2005.0313 PMC1626231 17148151

[9] Maldini D, Mazzuca L, Atkinson S (2005) Odontocete stranding patterns in the mainHawaiian Islands (1937–2002): how do they compare with live animal surveys? Pac Sci 59: 55–67.

[10] Norman S, Bowlby C, Brancato M, Calambokidis J, Duffield D, et al (2004) Cetacean strandings in Oregon and Washington between 1930 and 2002. J Cetac Res Manage 6: 87–100.

[11] Le Boeuf B, Perez-Cortes M H, Urban R J, Mate B, Ollervides u F (2000) High gray whale mortality and low recruitment in 1999: potential causes and implications. J Cetac Res Manage 2: 85–100.

[12] Scholin C, Gulland F, Doucette G, Benson S, Busman M, et al (2000) Mortality of sea lions along the central California coast linked to a toxic diatom bloom. Nature 403: 80–84. 10638756 10.1038/47481

[13] Doucette GJ, Cembella AD, Martin JL, Michaud J (2006) Paralytic shellfish poisoning (PSP) toxins in North Atlantic right whales *Eubalaena glacialis* and their zooplankton prey in the Bay of Fundy, Canada. Mar Ecol Prog Ser 306: 303–313.

[14] Taylor M (2003) Habitat degradation in the context of climate change: A review of recent work. International Whaling Comission. 12.

[15] MacLeod CD, Bannon SM, Pierce GJ, Schweder C, Learmonth JA, et al (2005) Climate change and the cetacean community of north-west Scotland. Biol Conserv 124: 477–483.

[16] Leeney RH, Amies R, Broderick AC, Witt MJ, Loveridge J, et al (2008) Spatio temporal analysis of cetacean strandings and bycatch in a UK fisheries hotspot. Biodivers Conserv 17: 2323–2338.

[17] Johnston DW, Bowers MT, Friedlaender AS, Lavigne DM (2012) The effects of climate change on harp seals (*Pagophilus groenlandicus).* . PloS ONE 7(1): e29158 DOI:10.1371/journal.pone.0029158. 22238591 10.1371/journal.pone.0029158 PMC3251559

[18] Stenseth NC, Mysterud A, Ottersen G, Hurrell JW, Chan KS, et al (2002) Ecological effects of climate fluctuations. Science 297: 1292–1296. 12193777 10.1126/science.1071281

[19] Forchhammer MC, Post E (2004) Using large-scale climate indices in climate change ecology studies. Popul Ecol 46: 1–12.

[20] Hemery G, D'Amico F, Castege I, Dupont B, D'Elbee J, et al (2008) Detecting the impact of oceano-climatic changes on marine ecosystems using a multivariate index: The case of the Bay of Biscay (North Atlantic-European Ocean). Glob Change Biol 14: 27–38.

[21] Savenkoff C, Castonguay M, Chabot D, Hammill MO, Bourdages H, et al (2007) Changes in the northern Gulf of St. Lawrence ecosystem estimated by inverse modelling: Evidence of a fishery-induced regime shift? Estuar Coast Shelf S 73: 711–724.

[22] Harvey M, Devine L (2008) Oceanographic conditions in the Estuary and the Gulf of St. Lawrence during 2007: zooplankton. Technical Report. DFO Science Advisory Secretariat Research Document 2008/037: 35pp.

[23] Galbraith PS, Chassé J, Gilbert D, Larouche P, Brickman P, et al (2011) Physical oceanographic conditions in the Gulf of St. Lawrence in 2010. Technical Report. DFO Science Advisory Secretariat Research Document 2011/045: 82pp.

[24] Koutitonsky V, Bugden G (1991) The physical oceanography of the Gulf of St. Lawrence: a review with emphasis on the synoptic variability of the motion. In: Therriault JC (ed) The Gulf of St Lawrence: small ocean or big estuary. Can Spec Pub l Fish Aquat Sci 113: 57–90.

[25] Marchand C, Simard Y, Gratton Y (1999) Concentration of capelin (*Mallotus villosus*) in tidal upwelling fronts at the head of the Laurentian Channel in the St. Lawrence estuary. Can J Fish Aquat Sci 56: 1832–1848.

[26] Cotté C, Simard Y (2005) Formation of dense krill patches under tidal forcing at whale feeding hot spots in the St. Lawrence Estuary. Mar Ecol Prog Ser 288: 199–210.

[27] Simard Y, Lavoie D (1999) The rich krill aggregation of the Saguenay–St. Lawrence Marine Park: hydroacoustic and geostatistical biomass estimates, structure, variability and significance for whales. Can J Fish Aquat Sci 56: 1182–1197.

[28] Galbraith PS (2006) Winter water masses in the Gulf of St. Lawrence. J Geophys Res (C Oceans) 111: C06022.

[29] Gilbert DBP (1997) Interannual variability (1948–1994) of the CIL core temperature in the Gulf of St. Lawrence. Can J Fish Aquat Sci 54 (Suppl.1).

[30] Cyr F, Bourgault D, Galbraith PS (2011) Interior versus boundary mixing of a cold intermediate layer. J Geophys Res (C Oceans) 116: C12029.

[31] Geraci JR, Lounsbury VJ (2005) Cetaceans - Mass strandings. In: Geraci JR, Lounsbury VJ, editors. Marine Mammals Ashore: A Field Guide for Strandings. Baltimore MD: National Aquarium in Baltimore. 113–127.

[32] Harvey M, Galbraith PS, Descroix A (2009) Vertical distribution and diel migration of macrozooplankton in the St. Lawrence marine system (Canada) in relation with the cold intermediate layer thermal properties. Prog Oceanogr 80: 1–21.

[33] Galbraith PS, Larouche P, Chasse J, Petrie B (2012) Sea-surface temperature in relation to air temperature in the Gulf of St. Lawrence: interdecadal variability and long term trends. Deep-Sea Res II (Top Stud Oceanogr) 77–80: 10–20.

[34] Blasco D, Levasseur M, Bonneau E, Gélinas R, et al (1998) Patterns of paralytic shellfish toxicity in the St. Lawrence region in relationship with the abundance and distribution of Alexandrium tamarense. Sci Mar 67: 261–278.

[35] Mitchell MR, Harrison G, Pauley K, Gagné A, Maillet G, et al.. (2002) Atlantic Zonal Monitoring Program sampling protocol. Can Tec Rep Hydrog Ocean Sci. 23.

[36] Mysak L, Ingram R, Wang J, Van Der Baaren A (1996) The anomalous sea-ice extent in Hudson Bay, Baffin Bay and the Labrador Sea during three simultaneous NAO and ENSO episodes. Atmos Ocean 34: 313–343.

[37] Hurrell JW (1995) Decadal trends in the North Atlantic Oscillation: regional temperatures and precipitation. Science 269: 676. 17758812 10.1126/science.269.5224.676

[38] Stern HL, Heide Jørgensen MP (2003) Trends and variability of sea ice in Baffin Bay and Davis Strait, 1953–2001. Polar Res 22: 11–18.

[39] Ferguson SH, Stirling I, McLoughlin P (2005) Climate change and ringed sea (*Phoca hispida*) recruitment in western Hudson Bay. Mar Mammal Sci 21: 121–135.

[40] Johnston DW, Friedlander AS, Torres LG, Lavigne DM (2005) Variation in ice cover on the East Coast of Canada, February-March, 1969–2006: Implications for harp and hooded seals. Climate Res 29: 209–222.

[41] R Development Core Team (2008) R: A Language and Environment for Statistical Computing. R Foundation for Statistical Computing, Vienna, Austria.

[42] Zar JH (1999) Biostatistical analysis. New Jersey: Prentice-Hall Inc. 663 p.

[43] Burnham KP, Anderson DR (2002) Model selection and multimodel inference: a practical information theoretic approach. Fort Collins: Springer. 485 p.

[44] Buisson L, Thuiller W, Lek S, Lim P, Grenouillet G (2008) Climate change hastens the turnover of stream fish assemblages. Glob Change Biol 14: 2232–2248.

[45] DFO (2007) A Review of Ice Conditions and Potential Impact on Harp Seal Neonatal Mortality in March 2007. DFO Canadian Science Advisory Secretariat Science Response 2007/008.

[46] DFO (2003) Proceedings of the workshop on the development of research priorities for the northwest Atlantic blue whale population. DFO Canadian Science Advisory Secretariat Proceeding Series 2003/031.

[47] Sears R, Williamson JM, Wenzel FW, Bérubé M, Gendron D, et al (2000) Photographic identification of the blue whale (*Balaenoptera musculus*) in the Gulf of St. Lawrence, Canada. Reports of the International Whaling Commission (Special Issue) 12: 335–342.

[48] Walther GR, Post E, Convey P, Menzel A, Parmesan C, et al (2002) Ecological responses to recent climate change. Nature 416: 389–395. 11919621 10.1038/416389a

[49] MacLeod CD, Bannon SM, Pierce GJ, Schweder C, Learmonth JA, et al (2005) Climate change and the cetacean community of north-west Scotland. Biol Conserv 124: 477–483.

[50] Plourde S, Winkler G, Joly P, St-Pierre J-F, Starr M (2011) Long-term seasonal and interannual variations of krill spawning in the lower St Lawrence estuary, Canada, 1979 2009. J Plankton Res 33(5): 703–714.

[51] Bowen WD, Siniff DB (1999) Distribution, population, biology, and feeding ecology of marine mammals. In: Reynolds JE ΙΙΙ, Rommel SA, editors. Biology of marine mammals. Washington, D.C. : Smithsonian Institution Press. 423–484.

[52] Higdon JW, Ferguson SH (2009) Loss of Arctic sea ice causing punctuated change in sightings of killer whales (*Orcinus orca*) over the past century. Ecol Appl 19: 1365–1375. 19688941 10.1890/07-1941.1

[53] Ferguson SH, Dueck L, Loseto LL, Luque SP (2010) Bowhead whale *Balaena mysticetus* seasonal selection of sea ice. Mar Ecol Prog Ser 411: 285–297.

[54] Lesage V, Gosselin J-F, Hammill M, Kingsley MCS, Lawson J (2007) Ecologically and Biologically Significant Areas (EBSAs) in the Estuary and Gulf of St. Lawrence: a marine mammal perspective. Technical Report. DFO Science Advisory Secretariat Research Document 2007/046: 92pp.

[55] McMahon CR, Burton HR (2005) Climate change and seal survival: evidence for environmentaly mediated changes in elephant seal, *Mirounga leonina*, pup survival. P Roy Soc B-Biol Sci 272: 923–928. 10.1098/rspb.2004.3038 PMC1564088 16024347

[56] Biuw M, Boehme L, Guinet C, Hindell M, Costa D, et al (2007) Variations in behavior and condition of a Southern Ocean top predator in relation to in situ oceanographic conditions. P Nat Acad Sci USA 104: 13705. 10.1073/pnas.0701121104 PMC1959446 17693555

[57] Weise AM, Levasseur M, Saucier FJ, Senneville S, Bonneau E, et al (2002) The link between precipitation, river runoff, and blooms of the toxic dinoflagellate *Alexandrium tamarense* in the St. Lawrence. Can J Fish Aquat Sci 59: 464–473.

[58] Fauchot J, Levasseur M, Roy S, Gagnon R, Weise AM (2005) Environmental factors controlling *Alexandrium tamarense* (Dinophyceae) growth rate during a red tide event in the St. Lawrence Estuary (Canada). J Phycol 41: 263–272.

[59] Doniol-Valcroze T, Berteaux D, Larouche P, Sears R (2007) Influence of thermal fronts on habitat selection by four rorqual whale species in the Gulf of St. Lawrence. Mar Ecol Prog Ser 335: 207–216.

[60] Bost C, Cotté C, Bailleul F, Cherel Y, Charrassin J, et al (2009) The importance of oceanographic fronts to marine birds and mammals of the southern oceans. J Marine Syst 78: 363–376.

[61] Moore JE, Barlow JP (2013) Declining Abundance of Beaked Whales (Family Ziphiidae) in the California Current Large Marine Ecosystem. PLoS ONE 8(1): e52770 DOI:10.1371/journal.pone.0052770. 23341907 10.1371/journal.pone.0052770 PMC3547055

[62] Nemiroff L, Wimmer T, Daoust PY, McAlpine DF (2010) Cetacean strandings in the Canadian maritime provinces, 1990–2008. Can Field Nat 124: 32–44.

[63] Flewelling LJ, Naar JP, Abbott JP, Baden DG, Barros NB, et al (2005) Red tides and marine mammal mortalities. Nature 435: 755–756. 15944690 10.1038/nature435755a PMC2659475

[64] Murase H, Matsuoka K, Ichii T, Nishiwaki S (2002) Relationship between the distribution of euphausiids and baleen whales in the Antarctic (35 degrees E-145 degrees W). Polar Biol 25: 135–145.

