# Supplementary material for: Marine Mammal Strandings and Environmental Changes: A 15-Year Study in the St. Lawrence Ecosystem
Source: PLoS One. 2013 Mar 27;8(3):e59311. doi: 10.1371/journal.pone.0059311 (PMC3609766; doi:10.1371/journal.pone.0059311)
Supplement: File S1 — File contains: Table S1. Species composition in the stranding database for the Estuary and the Gulf of St.-Lawrence, Québec, Canada, 1994–2008. This table provides an overview of all marine mammal species stranded in the study area. Age and sex classes for each species were grouped together. Table adapted from [54]. Table S2. Species composition of marine mammal strandings reported in the Estuary and the Gulf of St.-Lawrence, Québec, Canada, from 1994–2008 (N = 1193). Table S3. Model selection for multiple linear regressions including environmental parameters as predictors of the inter-annual variation in marine mammal stranding events in the Estuary and the Gulf of St. Lawrence, Québec, Canada, 1994–2008. For each model, we report the sample size (n), the number of parameters (k), the Akaike Information Criterion (AIC), the value relative to the model with the lowest AIC (ΔAIC), AIC weight (ωAIC) as well as the adjusted R2 (R2adj.). Models are ranked by their AIC and best models are shown in bold. (DOC) [file pone.0059311.s001.doc]

**Supporting Information**

**Table S1.** Species composition in the stranding database for the Estuary and the Gulf of St.-Lawrence, Québec, Canada, 1994-2008. This table provides an overview of all marine mammal species stranded in the study area. Age and sex classes for each species were grouped together. Table adapted from [54].

| Group | Common name | Species | Habitat use | Strategy | Occurrence |
| --- | --- | --- | --- | --- | --- |
| Mysticete | Blue whale | *Balaenoptera musculus* | Feeding | Migrant | Common |
|  | Fin whale | *Balaenoptera physalus* | Feeding | Migrant | Common |
|  | Minke whale | *Balaenoptera acutorostrata* | Feeding | Migrant | Common |
|  | Humpback whale | *Megaptera novaeangliae* | Feeding | Migrant | Common |
|  | North Atlantic right whale | *Eubalaena glacialis* | Feeding | Migrant | Rare |
| Odontocete | Beluga | *Delphinapterus leucas* | Feeding and breeding | Resident | Common |
|  | Sperm whale | *Physeter macrocephalus* | Feeding | Migrant | Common |
|  | Northern Bottlenose whale | *Hyperoodon ampullatus* | Feeding | Migrant | Rare |
|  | Harbour porpoise | *Phocoena phocoena* | Feeding | Migrant | Common |
|  | Long-Finned Pilot whale | *Globicephala melas* | Feeding | Migrant | Rare |
|  | Atlantic White-sided dolphin | *Lagenorhynchus acutus* | Feeding | Migrant | Common |
|  | Pygmy sperm whale | *Kogia breviceps* | Feeding | Migrant | Rare |
|  | Sowerby’s beaked whale | *Mesoplodon bidens* | Feeding | Migrant | Rare |
|  | Common dolphin | *Delphinus delphis* | Feeding | Migrant | Rare |
|  | Striped dolphin | *Stenella coeruleoalba* | Feeding | Migrant | Rare |
| Seal | Harbour seal | *Phoca vitulina concolor* | Feeding and breeding | Resident | Common |
|  | Grey seal | *Halichoerus grypus* | Feeding and breeding | Migrant | Common |
|  | Harp seal | *Pagophilus groenlandicus* | Breeding and pagophilic | Migrant | Common |
|  | Hooded seal | *Cystophora cristata* | Breeding and pagophilic | Migrant | Common |
|  | Bearded seal | *Erignathus barbatus* | Feeding | Migrant | Rare |

**Table S2.** Species composition of marine mammal strandings reported in the Estuary and the Gulf of St.-Lawrence, Québec, Canada, from 1994- 2008 (N = 1193).

| Species^a^ | Years | | | | | | | | | | | | | | | | | Total |
| --- | --- | --- | --- | --- | --- | --- | --- | --- | --- | --- | --- | --- | --- | --- | --- | --- | --- | --- |
|  | | 1994 | 1995 | 1996 | 1997 | 1998 | 1999 | 2000 | 2001 | 2002 | 2003 | 2004 | 2005 | 2006 | 2007 | 2008 |  | |
| **Mysticete** | |  |  |  |  |  |  |  |  |  |  |  |  |  |  |  |  | |
| *Balaenoptera acutorostrata** | | 1 | 2 | 4 |  | 4 | 5 | 6 | 9 | 6 | 2 | 7 | 1 | 6 | 5 | 3 | 61 | |
| *Balaenoptera physalus** | | 1 | 1 |  | 1 | 2 | 1 | 3 | 1 | 4 | 2 | 1 | 2 | 2 | 2 | 2 | 25 | |
| *Balaenoptera musculus* | |  |  | 1 |  |  |  | 1 | 1 | 1 |  | 1 | 1 |  |  | 1 | 7 | |
| *Megaptera novaeangliae* | |  |  |  |  |  |  |  |  |  | 1 |  |  | 1 |  |  | 2 | |
| *Eubalaena glacialis** | |  |  |  |  |  |  |  | 1 |  |  |  |  |  |  |  | 1 | |
| **Subtotal** | | 2 | 3 | 5 | 1 | 6 | 6 | 10 | 12 | 11 | 5 | 9 | 4 | 9 | 7 | 6 | 96 | |
| **Odontocete** | |  |  |  |  |  |  |  |  |  |  |  |  |  |  |  |  | |
| *Delphinapterus leucas** | | 14 | 13 | 9 | 13 | 12 | 16 | 11 | 12 | 16 | 15 | 19 | 8 | 9 | 15 | 23 | 205 | |
| *Phocoena phocoena** | | 3 | 1 |  | 3 | 6 | 6 | 11 | 17 | 22 | 12 | 13 | 12 | 12 | 11 | 19 | 148 | |
| *Lagenorhynchus acutus** | | 3 |  | 1 |  | 1 | 6 | 3 | 1 | 5 | 1 |  | 1 | 2 | 7 | 2 | 33 | |
| *Delphinus delphis** | |  |  |  |  |  |  |  |  |  |  |  | 1 | 1 |  |  | 2 | |
| *Stenella coeruleoalba** | |  |  |  |  |  |  |  |  |  |  |  |  |  |  | 1 | 1 | |
| *Globicephala melas** | |  |  |  | 1 |  |  |  |  | 1 |  |  |  | 1 | 1 | 2 | 6 | |
| *Hyperoodon ampullatus** | | 2 |  |  | 1 |  |  |  |  |  |  |  |  |  |  |  | 3 | |
| *Mesoplodon bidens** | |  |  |  |  |  |  |  |  |  |  |  |  | 1 |  |  | 1 | |
| *Physeter macrocephalus* | |  |  |  |  |  | 1 |  |  | 1 | 1 |  |  | 1 |  | 1 | 5 | |
| *Kogia breviceps** | |  |  |  |  |  |  |  | 1 |  |  |  |  |  |  |  | 1 | |
| **Subtotal** | | 22 | 14 | 10 | 18 | 19 | 29 | 25 | 31 | 45 | 29 | 32 | 22 | 27 | 34 | 48 | 405 | |
| Cetacea spp. | | 1 | 2 | 2 | 2 | 5 | 5 | 8 | 5 | 2 | 2 | 2 | 5 | 3 | 2 | 2 | 48 | |
| **Subtotal** | | 25 | 19 | 17 | 21 | 30 | 40 | 43 | 48 | 58 | 36 | 43 | 31 | 39 | 43 | 56 | 549 | |

| Species | Years | | | | | | | | | | | | | | | Total |
| --- | --- | --- | --- | --- | --- | --- | --- | --- | --- | --- | --- | --- | --- | --- | --- | --- |
|  | 1994 | 1995 | 1996 | 1997 | 1998 | 1999 | 2000 | 2001 | 2002 | 2003 | 2004 | 2005 | 2006 | 2007 | 2008 |  |
| **Pinniped** |  |  |  |  |  |  |  |  |  |  |  |  |  |  |  |  |
| *Phoca vitulina** | 2 |  | 1 | 5 | 1 | 6 | 5 | 6 | 10 | 7 | 8 | 6 | 6 | 3 | 14 | 80 |
| *Halichoerus grypus** |  | 1 | 3 | 3 | 2 | 3 | 3 | 1 | 7 | 4 | 1 | 6 | 5 | 3 | 38 | 80 |
| *Pagophilus groenlandicus** |  | 2 | 2 | 2 | 2 | 4 | 4 | 5 |  | 4 | 1 | 2 | 3 | 2 | 2 | 35 |
| *Cystophora cristata** | 3 |  | 3 | 1 |  | 8 | 2 | 7 | 2 |  |  | 8 | 28 |  | 2 | 64 |
| *Erignathus barbatus** |  |  |  |  |  |  |  |  |  |  |  |  |  |  | 1 | 1 |
| Seal spp. | 8 | 11 | 33 | 14 | 8 | 17 | 11 | 21 | 22 | 18 | 18 | 13 | 84 | 34 | 72 | 384 |
| **subtotal** | 13 | 14 | 42 | 25 | 13 | 38 | 25 | 40 | 41 | 33 | 28 | 35 | 126 | 42 | 129 | 644 |
| **Total** | 38 | 33 | 59 | 46 | 43 | 78 | 68 | 88 | 99 | 69 | 71 | 66 | 165 | 85 | 185 | 1193 |

^a^Those species denoted with an asterisk (*) were included in statistical analyses of environmental parameters. Rare species (see Table S1 in File S1) were combined into one group. Other species were excluded due to the small number strandings.

**Table S3.** Model selection for multiple linear regressions including environmental parameters as predictors of the inter-annual variation in marine mammal stranding events in the Estuary and the Gulf of St. Lawrence, Québec, Canada, 1994-2008. For each model, we report the sample size (n), the number of parameters (k), the Akaike Information Criterion (AIC), the value relative to the model with the lowest AIC (ΔAIC), AIC weight (ωAIC) as well as the adjusted R^2^ (R^2^adj.). Models are ranked by their AIC and best models are shown in bold.

| Species | Models | k | AIC | ∆AIC | ωAIC | R^2^ adj. |
| --- | --- | --- | --- | --- | --- | --- |
| *Delphinapterus leucas* | NAOw.t.1. + Vol0 + LogKrilla | 3 | 76,49 | 0.00 | 0.11 | 0.52 |
| (n=205) | **NAOw.t.1.+ FWR+ Vol0 + LogKrilla** | **4** | **76.74** | **0.25** | **0.10** | **0.53** |
|  | NAOw.t.1. + Vol0 + LogKrilla + LogTA | 4 | 76.92 | 0.43 | 0.09 | 0.53 |
|  | NAO + NAOw.t.1.+ Vol0 + LogKrilla | 4 | 77.14 | 0.65 | 0.08 | 0.52 |
|  | NAOw.t.1. + Vol0 + SST + LogKrilla + LogTA | 4 | 77.53 | 1.04 | 0.07 | 0.52 |
|  | NAO + NAOw.t.1. + Vol0 + LogKrilla + LogTA | 5 | 77.70 | 1.21 | 0.06 | 0.52 |
|  | NAOw.t.1. + FWR + Vol0 + LogKrilla + LogTA | 5 | 77.73 | 1.24 | 0.06 | 0.51 |
|  | NAOw.t.1. + Vol0 + SST + LogKrilla | 3 | 77.78 | 1.29 | 0.06 | 0.50 |
|  | NAOw.t.1. + LogKrilla + LogTA + ICEV | 4 | 77.89 | 1.40 | 0.06 | 0.50 |
|  | NAOw.t.1. + FWR + Vol0 + SST + LogKrilla | 5 | 77.96 | 1.47 | 0.05 | 0.51 |
|  | NAOw.t.1. + Vol0 + LogKrilla + LogTA + ICEV | 5 | 78.08 | 1.59 | 0.05 | 0.50 |
|  | NAOw.t.1. + Vol0 + LogKrilla + ICEV | 4 | 78.28 | 1.79 | 0.05 | 0.48 |
|  | NAOw.t.1. + SST + LogKrilla + LogTA + ICEV | 5 | 78.34 | 1.85 | 0.04 | 0.49 |
|  | NAOw.t.1. + FWR + Vol0 + SST + LogKrilla + LogTA | 6 | 78.37 | 1.88 | 0.04 | 0.50 |
|  | NAO + NAOw.t.1. + Vol0 + SST + LogKrilla + LogTA | 6 | 78.40 | 1.91 | 0.04 | 0.50 |
|  | NAO + NAOw.t.1. + Vol0 + SST + LogKrilla | 5 | 78.48 | 1.99 | 0.04 | 0.49 |
| *Phocoena phocoena* | **FWR + SST + LogTA** | **3** | **84.12** | **0.00** | **0.26** | **0.40** |
| (n=148) | FWR + LogTA | 2 | 84.89 | 0.77 | 0.24 | 0.34 |
|  | SST + LogTA | 2 | 85.57 | 1.45 | 0.15 | 0.31 |
|  | SST + FWR + LogTA + resICEV | 4 | 85.73 | 1.61 | 0.13 | 0.36 |
|  | SST + FWR + Vol 0 + LogTA | 4 | 85.80 | 1.68 | 0.12 | 0.35 |
|  | LogTA | 1 | 85.84 | 1.72 | 0.11 | 0.25 |
|  | FWR + SST + LogKrilla + LogTA | 4 | 85.88 | 1.76 | 0.11 | 0.36 |
| *Lagenorhynchus acutus* | NAO + NAO.t.1. | 2 | 62.41 | 0.00 | 0.20 | 0.40 |
| (n=33) | **NAO + NAO.t.1. + ICEV** | **3** | **62.98** | **0.57** | **0.23** | **0.41** |
|  | NAO + NAO.t.1. + FWR | 3 | 63.35 | 0.94 | 0.21 | 0.39 |
|  | NAO + NAO.t.1. + Vol0 | 3 | 63.56 | 1.15 | 0.21 | 0.38 |
|  | NAO + NAO.t.1. + SST | 3 | 63.84 | 1.43 | 0.16 | 0.37 |
|  | NAO + ICEV | 2 | 63.90 | 1.49 | 0.14 | 0.34 |
|  | NAO + NAO.t.1. + FWR + ICEV | 4 | 64.25 | 1.84 | 0.11 | 0.38 |
|  | NAO + NAO.t.1. + LogKrilla | 3 | 64.29 | 1.88 | 0.11 | 0.35 |
|  | NAO + NAO.t.1. + LogTA | 3 | 64.36 | 1.95 | 0.10 | 0.35 |
| *Halichoerus grypus* | **NAO + NAOw.t.1. + LogTA + ICEV** | **4** | **102.47** | **0.00** | **0.18** | **0.41** |
| (n=80) | NAO + NAOw.t.1. + ICEV | 3 | 102.52 | 0.05 | 0.17 | 0.39 |
|  | NAO + NAOw.t.1. + Vol0 + LogTA + ICEV | 5 | 103.64 | 1.17 | 0.10 | 0.38 |
|  | NAO + NAOw.t.1. + FWR + LogTA + ICEV | 5 | 103.72 | 1.25 | 0.10 | 0.38 |
|  | NAO + NAOw.t.1. + SST + ICEV | 4 | 103.93 | 1.46 | 0.09 | 0.35 |
|  | NAO + NAOw.t.1. + SST + LogTA + ICEV | 5 | 104.12 | 1.65 | 0.08 | 0.36 |
|  | NAO + NAOw.t.1. + LogKrilla + ICEV | 4 | 104.21 | 1.74 | 0.08 | 0.34 |
|  | NAO + NAOw.t.1. + LogKrilla + LogTA + ICEV | 5 | 104.24 | 1.77 | 0.07 | 0.35 |
|  | NAO + NAOw.t.1. + FWR + ICEV | 4 | 104.35 | 1.88 | 0.07 | 0.33 |
|  | NAO + NAOw.t.1. + Vol0 + ICEV | 4 | 104.43 | 1.96 | 0.07 | 0.33 |
| *Balaenoptera acutorostrata* | **NAO + NAO.t.1.+Vol0 + SST +LogKrilla+LogTA+ICEV** | **7** | **50.73** | **0.00** | **0.25** | **0.81** |
| (n=61) | NAO + NAO.t.1. + Vol0 + LogKrilla + LogTA + ICEV | 6 | 51.42 | 0.69 | 0.23 | 0.80 |
|  | NAO + LogTA + ICEV | 3 | 51.60 | 0.87 | 0.28 | 0.78 |
|  | NAO + FWR + LogTA + ICEV | 4 | 52.29 | 1.56 | 0.28 | 0.78 |
|  | NAO + Vol0 + LogTA + ICEV | 4 | 52.51 | 1.78 | 0.34 | 0.78 |
|  | NAO+NAO.t.1.+FWR+Vol0+SST+LogKrilla+LogTA+ICEV | 8 | 52.52 | 1.79 | 0.51 | 0.78 |
|  | NAO + SST + LogTA + ICEV | 4 | 52.64 | 1.91 | 1.00 | 0.78 |
| *Cystophora cristata* | FWR + Vol0 + SST | 3 | 91.61 | 0.00 | 0.23 | 0.61 |
| (n=64) | **FWR + Vol0 + SST + ICEV** | **4** | **92.72** | **1.11** | **0.13** | **0.59** |
|  | Vol0 + SST | 2 | 92.92 | 1.31 | 0.12 | 0.55 |
|  | NAOw.t.1. + FWR + Vol0 + SST | 4 | 93.03 | 1.42 | 0.11 | 0.59 |
|  | FWR + Vol0 + SST + LogTA | 4 | 93.04 | 1.43 | 0.11 | 0.59 |
|  | FWR + Vol0 + SST + LogTA + ICEV | 5 | 93.28 | 1.67 | 0.10 | 0.59 |
|  | FWR + Vol0 + SST + LogKrilla | 4 | 93.41 | 1.80 | 0.09 | 0.58 |
|  | NAO + FWR + Vol0 + SST | 4 | 93.60 | 1.99 | 0.09 | 0.57 |
| Log Seal mass stranding | NAOw.t.1. + ICEV | 2 | 45.38 | 0.00 | 0.24 | 0.39 |
| (n=4008) | **NAOw.t.1. + FWR + ICEV** | **3** | **45.69** | **0.31** | **0.20** | **0.40** |
|  | NAOw.t.1. + SST + ICEV | 3 | 46.57 | 1.19 | 0.13 | 0.36 |
|  | NAOw.t.1. + FWR + SST + ICEV | 4 | 46.76 | 1.38 | 0.12 | 0.38 |
|  | NAO + NAOw.t.1. + ICEV | 3 | 46.88 | 1.50 | 0.11 | 0.35 |
|  | NAOw.t.1.+LogKrilla+ICEV | 3 | 47.02 | 1.64 | 0.10 | 0.34 |
|  | NAOw.t.1. | 1 | 47.29 | 1.91 | 0.09 | 0.26 |
| Rare species | **NAOw.t.1. + FWR + Vol0 + LogTA + ICEV** | **5** | **44.41** | **0.00** | **0.39** | **0.34** |
| (n=15) | NAO + NAOw.t.1.+ FWR+Vol0 + LogTA + ICEV | 6 | 44.99 | 0.58 | 0.29 | 0.33 |
|  | NAOw.t.1. + FWR + Vol0 + SST + LogTA + ICEV | 6 | 46.02 | 1.62 | 0.17 | 0.28 |
|  | NAO + NAOw.t.1.+ Vol0 + LogTA + ICEV | 5 | 46.36 | 1.95 | 0.15 | 0.25 |
